# The Combination of Radiotherapy With Immunotherapy and Potential Predictive Biomarkers for Treatment of Non-Small Cell Lung Cancer Patients

**DOI:** 10.3389/fimmu.2021.723609

**Published:** 2021-09-21

**Authors:** Lu Meng, Jianfang Xu, Ying Ye, Yingying Wang, Shilan Luo, Xiaomei Gong

**Affiliations:** ^1^Department of Radiation Oncology, Shanghai Pulmonary Hospital, Tongji University School of Medicine, Shanghai, China; ^2^Department of Oncology, Shanghai Pulmonary Hospital, Tongji University School of Medicine, Shanghai, China

**Keywords:** non-small cell lung cancer (NSCLC), radiotherapy, immunotherapy, immune checkpoint inhibitor (ICI), biomarker

## Abstract

Radiotherapy is an effective local treatment modality of NSCLC. Its capabilities of eliminating tumor cells by inducing double strand DNA (dsDNA) damage and modulating anti-tumor immune response in irradiated and nonirradiated sites have been elucidated. The novel ICIs therapy has brought hope to patients resistant to traditional treatment methods, including radiotherapy. The integration of radiotherapy with immunotherapy has shown improved efficacy to control tumor progression and prolong survival in NSCLC. In this context, biomarkers that help choose the most effective treatment modality for individuals and avoid unnecessary toxicities caused by ineffective treatment are urgently needed. This article summarized the effects of radiation in the tumor immune microenvironment and the mechanisms involved. Outcomes of multiple clinical trials investigating immuno-radiotherapy were also discussed here. Furthermore, we outlined the emerging biomarkers for the efficacy of PD-1/PD-L1 blockades and radiation therapy and discussed their predictive value in NSCLC.

## 1 Introduction

Non-small cell lung cancer (NSCLC) is a prevalent disease with high morbidity and mortality, accounting for 85% of lung cancer cases. Most NSCLC patients are at late stages when diagnosed and lose the chance for tumor resection, with a low 5-year survival rate of approximately 24% (1). Therefore, it is imperative to explore new regimens and combination strategies to improve the survival prospects of NSCLC. With a remarkable development in recent years, radiotherapy has become an indispensable locally therapeutic strategy, applied to over 50% patients in different periods for treatment. Advanced technologies such as intensity-modulated radiation therapy (IMRT) and image-guided radiotherapy (IGRT) can deliver radiation rays to the delineated tumor target precisely with minor damage to the surrounding healthy tissues. However, resistance to radiotherapy often occurs and may lead to local, nodal, and distant recurrence. Moreover, increasing researchers has shed light on both of the influence of radiation therapy on immune tumor microenvironment. It is known irradiated tumor cells can serve as *in situ* vaccine that stimulates systemic adaptive immune response and promotes distant tumor regression. This phenomenon was first depicted by Mole RH et al. and is called the “abscopal effect”. Although the activated CD8^+^ T cells and other immune stimulative cells can migrate to and infiltrate metastasis sites, anti-tumor effects could be stifled by the upregulation of immune-suppressive cells, cytokines, and other molecules (2). The problems above indicate that radiotherapy alone is insufficient to eliminate both primary and metastatic tumor lesions, and exploring novel partners for treatment is imperative.

Immune checkpoints are surface proteins on T cells and other immune cells that can negatively regulate immune response activation to maintain self-tolerance by various antigens, including tumor antigens. PD-L1, expressed on tumor cells as well as tumor-infiltrating lymphocytes, can inhibit the proliferation of T cells and secretion of cytokine through binding to PD-1 and B7 receptors on activated immune cells (3). Cytotoxic T lymphocyte-associated protein 4 (CTLA-4) is another well characterized immune checkpoint that is primarily expressed on activated T cells and FoxP3^+^ regulatory T cells (Tregs) (4). It prevents the second costimulatory signal in T cell activation through binding to CD80/86 on APC competitively with a higher affinity than CD28 (5). CTLA-4 pathway also contributes to the immunosuppressive function of Tregs (6). Immune checkpoint inhibitors (ICIs) can enhance the intrinsic immune response against tumor antigens by removing the brake on T-cell activation and function. Immunotherapeutic agents that target immune checkpoint pathways have shown to be promising in clinical trials. Since the approval of ipilimumab (CTLA-4 monoclonal antibody) for metastatic melanoma in 2011, a variety of ICIs have been emerging, especially antibodies that target PD-1 and PD-L1. Some of them have been rapidly incorporated into the first or multiple line treatments, dramatically altering the treatment landscape of many solid tumors including malignant melanoma, NSCLC, renal cell carcinoma, bladder cancer and so on (7). Compared with CTLA-4 blockades, PD-1/PD-L1 blockades have shown efficacy in a wider range of cancer types and lower toxicity (8). As for metastatic NSCLC, nivolumab, pembrolizumab, and atezolizumab have been recommended in clinical practice for treatment. Notably, Pembrolizumab is recommended for monotherapy as first-line treatment for NSCLC patients without oncogenic driving mutations and with a high PD-L1 expression (tumor proportion score (TPS) ≥ 50%). However, the response rate to immune checkpoint blockade monotherapy has been disappointing in multiple clinical trials. Moreover, a range of immune-related toxicities such as colitis, hypophysitis, pneumonitis, thyroiditis, inflammatory arthritis, and more have been reported (9). Under these conditions, it has been recognized that combining anti-PD-1/PD-L1 treatment and radiation therapy could help overcome treatment resistance and improve survival benefits of patients synergistically. Studies on melanoma and NSCLC have accounted for a big part of studies relative to radioimmunotherapy. They carry a large mutation load and are characterized as high immunogenicity. Therefore, administration of radiation and ICIs to increase the tumor immunogenicity and immunoreactivity is critical in tumor regression.

Currently, delivery of radiotherapy and PD-1/PD-L1 blockades in the clinical practice is still imprecise, with a limited ability to identify patients who will get survival benefit from immune blockades and radiation treatments or patients who are likely to suffer from adverse effects caused by these modalities. For this reason, predictive biomarkers are in urgent need in order to tailor the treatment strategy for individuals. At present, only PD-L1 expression levels on tumor cells have been widely used as a standard predictor to drive anti-PD-1/PD-L1 treatment in the clinic, while multiple other markers detected by genomic, transcriptomic, proteomic, and metabolomic analysis are still under investigation and validation. This article will discuss the immune effects of radiotherapy in tumor microenvironment and the advances in applying radiotherapy plus immunotherapy. We will also outline the emerging biomarkers which show potential to predict response to PD-1/PD-L1 inhibitors as well as radiotherapy in NSCLC.

## 2 Radiotherapy Effects on Immune Tumor Microenvironment

**Figure 1:**
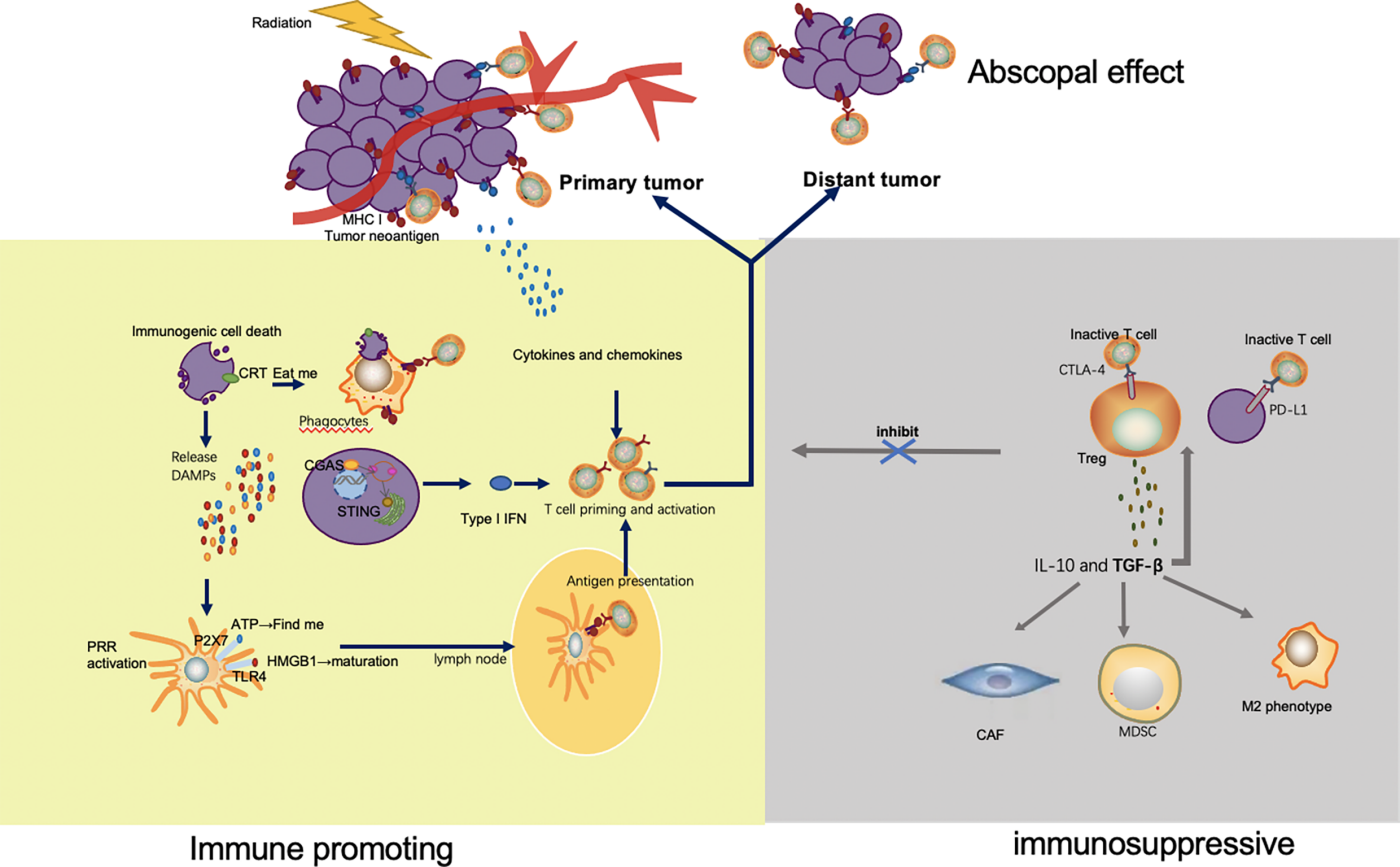
Mechanisms by which radiation enhances and inhibits immune response. Irradiation of tumor leads to immunogenic cell death and release of tumor neoantigens. Multiple DAMPs secreted from apoptotic tumor cells facilitate the uptake and presentation of neoantigens. Released ATP and exposure of CRT send the ‘find me’ and ‘eat me’ signal to APC and phagocyte respectively. HMGB1 promotes maturation of APC through binding with TLR4. As a result, APC migrates to lymph nodes and presents neoantigens to T cells mediated by MHC pathway. In addition to antigen presentation, type I IFN *via* cGAS-STING pathway and a variety of other cytokines and chemokines also take part in T cell priming and activation. The activated T cells, especially the CD8^+^ T cells proliferate, home to tumor, and exert their killing effect. Apart from the irradiated tumor site, effector T cells also migrate to distant tumors and drive abscopal effect. However, immune escape appears after radiation, which limits the efficacy of radiation. Upregulation of immune checkpoints, such as CTLA-4 on Tregs and PD-L1 on tumor cells, inhibits the activation and function of T cells. TGF β secreted by Tregs is capable to significantly increase the number of immunosuppressive cells in the TME, including Treg, CAF, MDSC and M2 phenotype macrophage.

Radiotherapy brings damage to target tumor cells through not only DNA strand break but also the killing effects of activated immune cells. Irradiation has been known to modulate the landscape of immune tumor micro-environment (TME) and systemic response both positively and negatively in a variety of ways (**Figure 1**).

### 2.1 Radiation Reprograms Immune TME

#### 2.1.1 Radiation Induces Immunogenic Cell Death

Immunogenic cell death (ICD) can be induced by radiation therapy in a dose-dependent way and is composed of the induction of organellar and cellular stress, accompanied by the release of neoantigen and endogenous danger signals-damage associated molecular patterns (DAMPs) from dying tumor cells, such as high-mobility group box 1 (HMGB1), adenosine triphosphate (ATP), as well as exposure of calreticulin (10, 11). Extracellular ATP was determined to be a critical ‘find me’signal in phagocytes recruitment through binding to P2Y2 receptor on apoptotic cells (12). It also promotes secretion of IL-1β and IL-18 (13). The translocation of calreticulin on cell membrane sends the important ‘eat me’ danger signal and induces clearance of tumor cells by antigen presenting cells(APC), such as dendritic cells(DCs) and phagocytic cells. HMGB1 enhances antigen processing and presentation and maturation of APC especially dendritic cells (DCs) after binding to specific pattern recognition receptors (PRRs), such as Toll like receptor-2 and Toll like receptor-4 (14, 15). Increased type I IFN *via* cytosolic DNA sensing pathway——cyclic guanosine monophosphate-adenosine monophosphate (cGAMP) synthase (cGAS) and its downstream effector Stimulator of Interferon Genes (STING) also plays a critical role in T cell activation and the drive of immune response after radiotherapy (16, 17). Radiotherapy effectuates the killing of tumor cells by DNA double strand break (DSB) induction, which leads to the mechanism by which self-DNA becomes exposed to the cytoplasm (18). Studies conducted by Deng et al. demonstrates that cytosolic DNA sensed by cGAS triggers IFN-I stimulated genes (ISG) induction and type I IFN secretion mainly by CD11c^+^DCs. They also determine that STING is critical to tumor control through radiotherapy *in vivo* (17).

#### 2.1.2 T Cell Infiltration and Activation

It is known that cytotoxic CD8^+^ T lymphocyte immunity is critical in the control of the tumor. Although radiotherapy has destructive effects on immune cells, recent studies have shown that a large proportion of T cells in irradiated tumor sites survived with increased motility and higher production of IFN γ compared with T cells in unirradiated tumors (19). T cell activation and priming could be elicited by radiotherapy through sufficient antigen and co-stimulation provided by local antigen presenting cells (APC) as well as chemokines secretion such as CXCL16 (20, 21). Numerous genes involved in antigen processing and presentation pathways were found to be upregulated in many cell lines after irradiation through transcriptome analysis (22). Reits et al. reported that radiotherapy could dose-dependently upregulate the expression of MHC-I molecules on neoplastic cells.

The increase of intracellular existing protein degradation leads to increased peptide pool, upregulated protein translation and peptide production through the kinase mammalian target of rapamycin (mTOR), and new antigen production by immunogenicity mutation, which are induced by irradiation (23, 24). In addition, fractionated radiotherapy leads to T cell repertoire broaden as well as expansion of preexisting polyclonal T-cell in the locally irradiated tumor (25–27). Consequently, ICIs may help further potentiate the systemic immune, enabling T cells to recognize more tumor antigen peptides.

#### 2.1.3 Immune-Suppressive Effects After Radiation

However, radiotherapy also elicits immunosuppressive signals such as the PD-1/PD-L1 and CTLA-4 pathway, restricting local and systemic anti-tumor immune effects. Mechanistic analysis suggested that IFN γ secreted by CD8^+^ T cells mediated upregulation of PD-L1 on tumor cells, thus inducing T cell exhaustion and weakening the tumor-antigen specific immune response (28, 29). This challenge could be overcome through combination with anti-PD-1/PD-L1 regimen to rescue T cell activity. In addition to PD-1 and CTLA-4 axis, regulatory CD4 ^+^ FOXP3 ^+^ T cells (Tregs) also increase after radiotherapy. Irradiated Tregs show elevated immunosuppressive capability than nonirradiated ones. Treg cells generate immunosuppressive transforming growth factor beta (TGFβ) and IL-10 through the CTLA4 signal pathway (30).

TGFβ in the TME is a potent suppressor of anti-tumor immune response. It not only promotes the recruitment and proliferation of immunosuppressive cells, including Tregs, cancer associated fibroblasts (CAF), myeloid suppressor cells (MDSCs), M2 macrophage phenotype, but also inhibit effects of CD8+ T lymphocytes and natural killer (NK) cells (31). Upregulation of these immune inhibitory cells contributes to the restricted efficacy of radiotherapy and immunotherapy (32, 33). Moreover, TGFβ plays a role in normal tissue toxicity especially chronic fibrosis after radiotherapy from a variety of pathways, leading to remodeling of lung architecture through promoting accumulation of collagen and myofibroblasts conversion (31). It is interesting to note that TGFβ contributes to radio-resistance of intra-tumoral T cells (19). Rube et al. found that higher dose of radiation (12Gy *vs.* 6Gy) was related to more significant release of TGFβ, reaching the summit after 12 hours and returning to basal level subsequently within one week (34). Similarly, delivery of low-dose radiation has been reported to repolarize macrophages from M2- to the pro-immunogenic M1-phenotype and enhancing infiltration of NK cells (35, 36). Therefore, radiation dose is a critical factor that influences tumor control as well as tissue toxicity post radiotherapy.

### 2.2 The Abscopal Effect

Abscopal effects have been known as the observation of both local and distant tumor control after applying irradiation to the regional tumor target. Accumulated evidence supports the hypothesis that irradiated tumor cells may act as an “*in situ* vaccine” through the production of tumor-associated antigens, induction of DAMPs, and modulation of the tumor microenvironment. Both preclinical and clinical studies showed that IFN β induced by cGAS-STING pathway after radiation played a key role in distant tumor regression (37, 38). The exposure of immunogenic mutation on tumor cells mediated by radiotherapy is another mechanism that contributes to a robust systemic T cell response and occurrence of abscopal effects (38). However, the occurrence of abscopal effect is rare and limited to case report. Therefore, radiotherapy alone is not enough to drive abscopal effect.

Demaria et al. reported 2 patients responding to the combination therapy of radiation and ICI when they failed with chemotherapy or anti-CTLA-4 therapy. It indicates that radiotherapy has a potential to drive the regression of distant tumors together with immunotherapy (38). The combination of radiotherapy and ICIs has shown to improve the opportunity to boost abscopal response rates, ranging from 23% to 38%, compared with immunotherapy or radiotherapy alone in multiple clinical trials (38–43). In addition, a volume of preclinical studies added immune-stimulative drug to radiotherapy, such as immunoadjuvant FMS-like tyrosine kinase receptor 3 ligand (sFLT3L) (44), DCs (45), granulocyte-macrophage colony-stimulating factor (GM-CSF) (46), CTLA-4 blockades (47, 48), PD-1/PD-L1 blockades (2, 25). Despite the delivered irradiation dose and fraction as well as types of immune enhancers varied, most combination therapy groups in mice models with established tumors derived from different cell lines demonstrated improved survival and distal tumor regression compared with mono-radiotherapy groups. Therefore, we hypothesize that immuno-radiotherapy has the potential to transfer rare abscopal events to a therapeutic effect.

## 3 Combination of Radiotherapy and Immunotherapy in NSCLC

### 3.1 Clinical Trials Combing RT and ICIs

Radiotherapy has emerged as a promising partner of immunotherapy due to its ability to modulate the tumor microenvironment and elicit immune responses. Increasing prospective and retrospective studies have investigated the impacts of radiation plus anti-PD-1/PD-L1 therapies on patient survival, tumor control, abscopal effects, and toxicities in different clinical scenarios (**Table 1**). Stereotactic ablative radiotherapy (SABR) is now considered the standard treatment in early-stage (stage I and stage IIA) NSCLC patients who are unfit or unwilling to accept surgery (54). SABR is characterized by accurate delivery of high-dose radiation to the target area with hypofractionation and short treatment time (55). Despite the high local control and less toxicity with SABR, the risk of distant recurrence (20%-25%) prompts ongoing trials to assess the efficacy of adjuvant PD-1/PD-L1 blockades after SABR (56). As for locally advanced NSCLC, many novel approaches were investigated as consolidation therapy to improve the efficacy of platinum-based chemoradiotherapy (CRT), but most of them failed. The PACIFIC trial showed a striking survival benefit for the addition of durvalumab to definitive CRT in stage III unresectable NSCLC patients. This randomized phase 3 trial observed an improved 24 month-OS rate (66.3% *vs.* 55.6%) and prolonged PFS duration (17.2 months *vs.* 5.6 months) in the durvalumab group compared with the placebo group. The time for distant metastasis was also delayed with adjuvant durvalumab treatment (28.3 months *vs.* 16.2 months). Interestingly, the improved outcomes were independent of PD-L1 status (49, 50). These results greatly encouraged the promise of administrating ICI as consolidation treatment to enhance the efficacy of local CRT in unresectable stage III patients.

**Table 1:** Prospective clinical trials evaluating PD-1/PD-L1 blockades combining with RT in NSCLC.

Trial	Phase	Stage	N	sequence	ICI Agent	RT Dose	OS	PFS	Toxicity≥G3(%)
**PACIFIC** (49, 50)	III	Locally advanced Unresectable III	713	CRT> ICI *vs.* CRT>placebo	Durvalumab (10 mg per kilogram of body weight intravenously every 2 weeks)	54-66Gy	1-yr: 83.1% *vs.*74.6%	Median 17.2 *vs.* 5.6 months	30.5% *vs.* 26.1%
							2-yr: 66.3% *vs.* 55.3%		
**HCRN LUN 14 -179** (51)	II	Locally advanced Unresectable IIIA/B	92	CRT> ICI	Pembrolizumab (200 mg intravenously every 3 weeks)	59.4-66.6Gy	1-yr: 81.2%	Median 18.7months	4.30%
							2-yr: 62.0%		
							3-yr: 48.5%		
**DETERRED** (52)	II	Locally advanced Unresectable III	40	CRT+ICI>CT+ICI *vs.* CRT>CT+ICI	atezolizumab (1200 mg IV Q3 weeks)	60-66Gy/30-33 fractions	1-yr: 60% *vs.* 77%	Median 20.1 months *vs.* NR 1-yr:60% *vs.* 66%	57%
**ETOP NICOLAS** (53)	II	Locally advanced Unresectable IIIA/B	79	CRT+ICI> ICI	Nivolumab (360 mg every three weeks for the first four doses, followed by 480 mg every four weeks)	66 Gy/33fractions	NR	NR	10% for pneumonitis
**PEMBRO -RT** (42)	II	metastatic IV	74	SBRT (single tumor site)> ICI *vs.* ICI	Pembrolizumab (200mg every three weeks)	24Gy/3fractions	Median 15.9 *vs.* 7.6months	Median 6.6 *vs.* 1.9months	17% *vs.* 22%

RT, radiotherapy; CRT, chemoradiotherapy; CT, chemotherapy; SBRT, stereotactic body radiotherapy; ICI, immune checkpoint inhibitors; ORR, overall response rate; OS, overall survival; PFS, progression-free survival; NR, not reported.

Apart from the validated benefits from adjuvant ICI therapy after CRT, the safety profile of immuno-radiotherapy as part of definitive therapy or neoadjuvant therapy in locally advanced NSCLC has also been confirmed and its influence on patient survival will be conducted in larger cohorts (52, 53). Similarly, clinical benefits from the combination of ICI and radiotherapy in metastatic NSCLC have been validated in preclinical and clinical evidence. Combination of immunotherapy and SABR (I-SABR) can reactivate the immune system of patients undergone progression to ICI monotherapy and thus enhancing patients’ distant tumor control and clinical benefits (43). A randomized multicenter phase 2 study (PEMBRO-RT) enrolled 92 patients with advanced NSCLC and divided them into experiment arm (Pembrolizumab after SBRT of 8 Gy×3) and control arm (Pembrolizumab alone). Although the increase in response rate and survival benefits were insignificant in the experiment arm compared with the control arm, the value of PFS and OS multiplied, especially in patients with negative PD-L1 expression (42).

### 3.2 Exploration of the Optimal Combination Scheme

The positive results of immuno-radiotherapy have prompted researchers to explore the optimal combination schedule, including the radiation dose and fractionation, the sequence for radiotherapy and ICI administration, as well as the number of irradiated targets. Currently, a wide range of doses and fractions are used in clinical trials, and the consensus about the best schedule has not been determined yet. Generally higher dose in SABR seems to activate a more robust systemic immune response and associate with a higher response rate of unirradiated lesions compared with conventionally fractionated radiotherapy (40, 41). However, the single dose of radiation higher than 12 to 18 Gy was demonstrated to hinder anti-tumor T-cell response stimulated by IFNβ because of the dose-dependent upregulation of nuclease TREX1. Overexpressed TREX1 would degrade cytosolic dsDNA remarkedly, which became an obstacle of activating the cGAS-STING pathway as well as the abscopal effect (37). Many other studies also showed better control of distant tumors in hypofractionation rather than single-dose ablative radiotherapy (48, 57). However, preclinical and clinical studies completed by James Welsh et al. indicate that it is promising to combine the low-dose radiation of 1 Gy fraction to metastatic tumor lesions, with the high-dose radiation to the primary tumor lesions in order to further drive abscopal effects. This novel radiation strategy that is called ‘RadScopal’ technique could be a preferred partner of immunotherapy, compared with high-dose or low-dose radiation alone (36, 58, 59). The mechanisms underlying the positive results involve modulation of the immune-inhibitory tumor stroma such as TGF-β down-regulation and M1 macrophage polarization, and promote infiltration and activation of anti-tumor effective cells such as NK and CD4+ T cells. Relative prospective clinical trials are ongoing to confirm the effects of low-dose radiation.

The most appropriate time window for the combination therapy has not been determined yet. Administration of ICIs before or concurrent with radiotherapy is preferred in multiple preclinical and clinical trials (28, 60). A subgroup analysis of the PACIFIC trial suggested that delivery of ICI within 14 days after irradiation could obtain improved survival benefits in NSCLC. It is confusing that the delayed immunotherapy post radiotherapy was also effective in other studies (51, 61, 62). In a case report by Postow et al., the patient who received ipilimumab 2 months after palliative radiotherapy showed both primary and distant tumor regression significantly (62). Preliminary data from DETERRED showed that combing ICI with radiotherapy concurrently rather than sequentially might better enhance the synergistic effect of radiotherapy and immunotherapy (52), but the strong killing effect of radiation on lymphocytes must be taken into consideration. Whether this will affect subsequent immunotherapy or whether concurrent chemoradiotherapy combined with triple therapy of immunity will bring more serious side effects, especially the occurrence of radioactive pneumonia, remains to be further explored.

Other factors such as the volume and number of targets also need to be further explored. Irradiation on the tumor-draining lymph node was found to reduce the CD8^+^ and stem-like CD8^+^ T-cell and therefore hinder the distant tumor elimination (63). Furthermore, multisite or all sites irradiation can increase the release and presentation of tumor-associated antigens and improve patients’ clinical outcomes compared with single-site irradiation, but radiation-related toxicities need to be controlled in a tolerable extend (64).

To date, most of the studies in terms of combination therapy are retrospective or with small-sized cohorts. Robust evidence for the optimal scheme of immuno-radiotherapy is required in larger randomized clinical trials with minimized heterogeneity among patients.

## 4 Predictive Biomarkers of Immuno-Radiotherapy Efficacy

Considering the improved survival benefits for responders and the unnecessary adverse effects and costs for non-responders, developing predictive biomarkers to help physicians choose the most suitable therapeutic scheme to individuals is imperative. We have summarized the emerging biomarkers that have a potential to stratify patients into groups with different responses and risk levels in clinical practice (**Table 2**).

**Table 2:** Biomarkers of Radiotherapy and Immunotherapy Efficacy in NSCLC.

Biomarker		Sample origin	Treatment	Results	References
↑PD-L1		FFPE/surgical specimen	ICIs	↑ OS, PFS	(65–67)
TILs	↑CD8^+^ TIL	FFPE	CCRT	↑PFS, OS	(68, 69)
	↑TIL density≥10%	H&E–stained sections	nivolumab	↑ survival	(70)
	↑PD-1 ^+^CD8^+^T cells	fresh tumor specimens	durvalumab	↑ORR, OS, PFS	(71)
	↓PD-1 ^+^CD8^+^T cells	FFPE	nivolumab	↑OS, DFS and response	(72)
	↑CD8×PD-L1 signature	FFPE	durvalumab	↑response	(73)
	↑PD-L1^high^ Tregs	peripheral blood, FFPE from surgery and biopsy	pembrolizumab/nivolumab	↑response, PFS	(71)
Immune related gene expression profiling (GEP)	↑18-gene T cell–inflamed GEP	FFPE	pembrolizumab	↑response rates, PFS	(74)
	↑4-gene IFN γ positive (IFN γ+) signature	FFPE/frozen biopsies	durvalumab	↑ORR, median PFS and OS	(75)
	Profile of 24 chemokines and immunosuppressive molecules	FFPE	pembrolizumab	differentiate responders from non-responders, with predictive correlation up to 85.0%	(76)
	↑antigen processing machinery (APM) score	FFPE	ICIs	↑DFS, OS	(77)
	↓EMT (more epithelial)/↑ Inflammation signature score	FFPE	ICIs	↑response, PFS, OS	(78)
Radiosensitivity signature (RSS)	31 gene-signature ( PD-L1-high-RR group)	NCl-60	RT	↓survival (Not validated in NSCLC)	(79–81)
	RSS+IMS (radiation-sensitive + immune-effective)	fresh frozen tumors	RT	↑DSS, response (Not validated in NSCLC)	(82)
↑TMB		FFPE/surgical specimen	ICIs	↑response	(83–85)
		FFPE	RT	↑durable survival	(86)
Peripheral blood cell and lymphocyte ratios	↑ALC	full blood	RT	↑Abscopal effect	(87)
	↑ALC	full blood	Nivolumab	↑PFS, OS	(88)
	↑PD-1+ CD8+ T cells	full blood	pembrolizumab	↑RR, PFS, OS	(89)
			nivolumab		
			atezolizumab		
	↑CD56^+^ NK	full blood	ICIs	↑response	(90)
	↑pre-NLR> 3.6	full blood	SBRT	↑mortality	(91)
	↑post-NLR>6	full blood	SRS	↓OS	(92)
	Poor LIPI (pre-dNLR >3+LDH >upper limit of normal )	full blood	Atezolizumab/nivolumab	↓OS, PFS, DCR	(93–95)
	NLR-low/TMB-high	full blood		↓OS, PFS, response rate	(96)
ctDNA	↓ctDNA	full blood	ICIs	↑PFS, OS	(97, 98)
	cfDNA >20% at the sixth week	full blood	nivolumab	↓OS, TTP	(99)
miRNA signature classifier (MSC)	↑post-NLR>6	full blood	SRS	↓OS	(92)
	Poor LIPI (pre-dNLR >3+LDH >upper limit of normal )	full blood	Atezolizumab/nivolumab	↓OS, PFS, DCR	(93–95)
	NLR-low/TMB-high	full blood		↓OS, PFS, response rate	(96)
	PD-L1+immune-related MSC risk level	Plasma and tissue samples	ICIs	identify the subgroup of patients with the worst ORR, PFS, OS	(100)
Imaging Biomarkers	↑pre-TMTV> 75 cm^3^+ pre-dNLR > 3	PET/CT	ICIs	↓OS, DCB	(101)
	↑IMPI=2: post-NLR < 4.9+ post-TLG < 541.5 (at the first restaging during ICI treatment )	PET/CT	ICIs	↓OS, PFS	(102)
	↓pre-SUV_max_ <5, pre-SUV_mean_<3.5	PET/CT	SABR	↑complete response at 6 months	(103)
	↑pre-MTV, pre-TLG	PET/CT	high-dose RT	↓OS	(104)
	↓ΔTLG-TN	PET/CT	RT	↓3 year-LCR, DSS	(105)
	↑pre-textural feature dissimilarity	PET/CT	SBRT	↑DSS, DFS (cut point: 18 and 18.4 respectively)	(106)
	↑radiomic features (model 1: Information Correlation 2 from PET+ flatness from CT; model 2: Information Correlation 2 and strength from PET)	PET/CT	SBRT	↓ local control	(107)

FFPE, formalin-fixed paraffin-embedded; ICIs, immune checkpoint inhibitors; RT, radiotherapy; CCRT, concurrent chemoradiotherapy; SBRT, stereotactic Body Radiotherapy; SABR, stereotactic ablative radiotherapy; SRS, stereotactic radiosurgery; OS, overall survival; PFS, progression free survival; TTP, time to progression; DFS, disease free survival; DCB, disease clinical benefit; ORR, overall response rates; DSS, disease specific survival; DCR, disease control rate; LCR, local control rate; EMT, epithelial mesenchymal transition; NCl-60, National Cancer Institute panel of 60 cell lines; SF2, survival fraction at 2 Gy; IMPI, immune-metabolic-prognostic index, ↑ increased; ↓ decreased.

### 4.1 PD-L1

Currently, tumor PD-L1 expression level is the only approved biomarker to drive ICIs therapy in clinical practice (108). A good correlation between higher tumor PD-L1 expression and more significant survival benefits from anti-PD-1/PD-L1 therapy has been validated in many clinical trials. KEYNOTE-024 and KEYNOTE-042 trials, which compared pembrolizumab with platinum-based chemotherapy in previously untreated, PD-L1-expressing advanced NSCLC, showed that the best survival benefit appeared in patients who had a PD-L1 TPS ≥ 50% rather than those with PD-L1 TPS ≥ 20% and ≥ 1% (65, 66). Outcomes from CheckMate-057 also confirmed the enhanced survival benefit with higher PD-L1 expression in advanced NSCLC patients treated with Nivolumab after the failure of chemotherapy (67). However, PD-L1 is not a perfect biomarker to predict response to PD-1/PD-L1 inhibitors. Some recent randomized trials showed no significant association of PD-L1 with clinical response to anti-PD-1/PD-L1 monotherapy and anti-PD-1/PD-L1 plus chemotherapy or chemoradiotherapy in NSCLC (67, 109, 110).

The role of tumor cell PD-L1 expression level in predicting the efficacy of radiotherapy or chemoradiotherapy in various cancer types has been investigated either. Higher baseline PD-L1 level was associated with poor response and prognosis in NSCLC patients treated with chemoradiotherapy (111, 112). Moreover, the significant benefit from the addition of SBRT before immunotherapy was only seen in the PD-L1 negative subgroup in the PEMBRO-RT study (42). However, it is disappointing that patients with PD-L1 expression levels lower than 25% obtain similar survival benefits to patients with higher PD-L1 levels in the PACIFIC study (50). Therefore, it is hard to confirm the predictive role of PD-L1 in NSCLC undergoing chemoradiotherapy alone or plus immunotherapy, considering the limited number of studies and conflicting results.

The heterogeneous expression of PD-L1 across time and space (113, 114), difference in sensibility among antibodies for PD-L1 IHC testing (115), and debatable threshold for PD-L1 expression assessment may account for the conflicting results above. Therefore, researches in larger cohorts are needed to standardize the sampling method, cut point, and companion antibodies for PD-L1 assessment.

### 4.2 Tumor Infiltrating Lymphocytes

Immune checkpoint inhibitors require adequate lymphocyte infiltration in tumor tissue in order to exert anti-tumor effects. Radiation can stimulate local innate and adaptive immune responses by increasing TIL. Therefore, the characteristics of TIL, including composition, density, functional state, and organization, could be used as predictors of response to radiotherapy and immunotherapy (116). Cytotoxic T cell is critical to tumor elimination and metastasis, which partly account for the positive association of CD8^+^T cell infiltrating with improved prognosis. An immuno-score determined by both CD3^+^ and cytotoxic CD8^+^ T cells densities in two locations—tumor section and invasive margin based on IHC was developed with a higher score representing the low risk of recurrence and improved survival benefits (117). In addition, TIL density over ≥10% could predict better survival benefits from ICIs (70). Higher level of PD-1+CD8+T cells in TME has been proved to predict better outcomes in NSCLC treated with PD-1/PD-L1 inhibitors (71). However, Results from a retrospective study conducted by Mazzaschi et al. showed that low density of PD-1^+^CD8^+^T cells predicted prolonged OS and DFS and response to nivolumab in NSCLC (72). Therefore, the association between PD-1^+^CD8^+^T cells and clinical outcome after ICIs treatment has not reached a consensus. A CD8xPD-L1 signature based on image analysis showed addictive power to the densities of CD8^+^ or PD-L1 alone as well as tumor cell PD-L1 expression level to predict the response to durvalumab therapy (73). Concomitantly, the enrichment of myeloid-derived suppressor cell (MDSCs), regulatory T cells (Tregs), tumor-associated macrophages, and neutrophils negatively impacts prognosis because of their inhibitory function on the immune response (118, 119). For example, a high frequency of PD-L1^+^ CD4^+^ CD25^+^Tregs in TME can predict improved response to ICI and PFS in NSCLC (71). In addition, German Corredor et al. established a computationally derived signature–Spatial TIL. The spatial patterns and arrangement of TILs were correlated with the risk of recurrence in NSCLC of early stages (120). In terms of radiotherapy, CD8^+^ TIL was significantly correlated with favorable clinical outcomes (68, 69).

Of note, the prognostic value of TILs differs according to histologic types, smoking habits, and mutation landscape (121). Although the presence of TILs is essential, the direct evaluation of TILs remains challenging due to the small histologic material derived from the biopsy and varied microenvironment in different metastases. Therefore, ongoing studies are trying to create and validate guidelines for TIL assessment.

### 4.3 Gene Expression Profiling

#### 4.3.1 Tumor Inflammation Signatures

Recently, multiple studies found that gene expression analysis related to tumor microenvironment through RNA isolated from formalin-fixed paraffin-embedded (FFPE) samples from patients before treatment with PD-1/PD-L1 blockades was predictive of treatment outcome in various cancer types (74, 75, 122, 123). Mark Ayers et al. developed T cell inflamed Gene Expression Profiles (GEPs) of 18 genes, representing a T cell–inflamed microenvironment and characterized by active IFN-γ signaling, T cell cytolytic activity, antigen presentation, chemokine production, and adaptive resistance. This profile was identified based on the Nano String nCounter gene expression system (NanoString Technologies, Inc., Seattle, WA) and conducted rigorous stepwise validation. Meanwhile, it demonstrated strong predictive value over clinical outcomes in a wide variety of solid tumors treated with pembrolizumab (124). Although TIS scores were higher in immunogenic tumor types, they were more variable within than between tumors types. Thus, a subset of patients who possess higher TIS scores is more likely to respond to anti-PD-1 blockade within any tumor type (123). Notably, this 18 gene profile performed favorably compared with PD-L1 immunohistochemistry (IHC) in a group of PD-L1-unselected patients, and one possible explanation for its better performance is that T-cell inflamed GEP measures multiple microenvironmental features which may influence induction and function of IFN-γ rather than a single analyte (124).

As expected, the correlations of T cell inflamed GEP with PD-L1 expression with TMB were low when analyzed in the Cancer Genome Atlas (TCGA). Meanwhile, each of the three biomarkers showed predictive value individually. Furthermore, tumors with high levels of both inflammatory and mutational biomarkers were most likely to derive clinical efficacy (74, 122, 123). Moreover, in tumor types with limited variability of TMB, TIS may provide potent predictive power on patient stratification (123). The outcomes of analyzing GEP in different aspects suggested that T cell inflamed GEP may function as a complementary predictor of response to anti-PD-1 treatment. Therefore, combining the two biomarkers may increase the accuracy of identifying populations who can potentially benefit from ICIs.

In addition to the 18 gene-T cell inflamed profile, researchers also identified other signatures of immune-related genes for NSCLC (75, 125). Brandon W et al. defined a four-gene IFNγ positive (IFNγ^+^) signature comprising IFNγ, CD274, LAG3, and CXCL9 through RNA sequencing of biopsies from advanced-stage NSCLC treated with durvalumab. The high signature patients showed improved survival benefits irrespectively of PD-L1 expression level (75). A profile of 24 chemokines and immunosuppressive molecules have been identified and validated, using the dataset of NSCLC patients treated with pembrolizumab from Rizvi et al. study (76). Addition of 23 chemokine and immune-suppressive molecule expression profiles to a PD-L1 profile raised the predictive correlation to 85.0% among NSCLC responders (76). Furthermore, some gene profiles that target specific immune mechanisms such as antigen processing and presentation and epithelial-to-mesenchymal transition (EMT) have been proved to be valuable predictors of ICB treatment in lung cancer (77, 78).

These patient-specific profiles of tumors defined above are used in investigations only and still under clinical validation at present. These multigene signatures can characterize TME and immune-related mechanisms comprehensively, which indicates the tumor’s immune status and thus holds a promise for identifying responding patients of ICI treatments.

#### 4.3.2 Radiosensitivity Gene Signatures

Exploration of radiosensitivity at the genome level has attracted much attention. An early radiosensitivity gene signature, including three known genes and one unknown gene, which was RbAp48, was developed by Torres-Roca JF et al. This radiation sensitivity classifier correctly predicted survival fraction at 2 Gy (SF2) of a high proportion (22 of 35) of tumor cell lines from the National Cancer Institute panel of 60 (NCI 60). Further analysis showed that overexpression of RbAp48 in HS-578T cell line increased the proportion of cells in the G2-M phase of cell cycle and was correlated with dephosphorylation of Akt, which suggests that RbAp48 may sensitize tumor cells through antagonizing Ras (126). A similar gene signature has been developed by Kim et al., including 31 genes. The radiation-related functions of these genes are involved in the cell cycle and DNA replication, cell junction, and cell adhesion. Through gene set analysis, these genes were found to be over-presented in the integrin, VEGF, phosphatidylinositol, MAPK, JAK-STAT, Wnt, and p53 signaling pathways (79). It was found that patients with high PD-L1 were more common in the radioresistant (RR) group than radiosensitive (RS) group, and the clinical outcome of the PD-L1-high-RR group was worse than other groups (80, 81). Therefore, integrating the 31-gene signature and PD-L1 expression status can help classify patient populations who may benefit most from the combination of radiotherapy and PD-1/PD-L1 blockade in clinic (80, 81, 127). In addition to focusing on the intrinsic tumor radiosensitivity only, integrating radiosensitivity signature and immune signature could significantly interact with radiotherapy. For example, the independent predictors RSS (radiosensitivity gene signature) and IMS (antigen processing and presentation-based immune signature) were developed by Cui et al. in radiation-treated breast cancer patients. When integrating both signatures, individuals in the radiation-sensitive and immune-effective group showed prolonged survival. In contrast, in the radiation-resistant + immune-defective group and either radiation-resistant or immune-defective group, a reverse trend and no significant differences were observed (82).

Stratifying patients for radiotherapy by performance on these gene signatures is still in its infancy and needs to be confirmed in future retrospective and prospective studies. Furthermore, radiation dose adjustment based on radiosensitivity gene signatures has been under investigation, predicting the most effective dose for delivery of personalized treatment plans, thus helping spare unnecessary radiotoxicity (128). Of note, tissue of origin could influence the expression of genes involved in these signatures. For example, in a10 hub genes model, radio-resistance induced by knockdown of c-Jun is mainly found in lung cell lines (129). Therefore, signatures validated as efficient predictors in other certain tumor types may not be valuable in NSCLC.

### 4.4 Tumor Mutational Burden

Tumor Mutational Burden is defined as the total number of nonsynonymous mutations, including base substitutions and short insertions/deletions, per coding area of a tumor genome and calculated as mutations per DNA Megabase (Mb). A high TMB can enhance tumor immunogenicity by inducing the production of tumor-associated neoantigen, which can be recognized by T cells and promote the activation of immune attacks (83). The relation between higher TMB and better response to anti-PD-1 or PD-L1 therapy has been demonstrated in multiple clinical trials (83–85). In addition, TMB can act as an independent predictive biomarker and does not correlate with tumor PD-L1 expression level in many trials which compared the efficacy of immunotherapy with chemotherapy (84, 85). However, CheckMate026 found that the overall survival (OS) of patients treated with nivolumab in the subgroup of high TMB was comparable with chemotherapy, which questioned the predictive role of TMB (84). Although the high rate of subsequent nivolumab use (68% of patients) in the chemotherapy group may contribute to that controversial result, further investigations need to confirm the predictive role of TMB in ICI therapy and explore the additive value of combining TMB with other markers.

The role of TMB in predicting radiotherapy efficacy remains uncertain. In many clinical studies of immuno-radiotherapy in NSCLC, the level of TMB in the population was not obtained. High TMB was demonstrated to correlate with poor outcome in head and neck squamous cell carcinoma (HNSCC) patients (130), however, a group of irradiated NSCLC patients with higher TMB showed durable survival, and this benefit was not observed in the nonirradiated patient group in another study (86). In terms of immune-radiotherapy, TMB could not serve as a well-qualified predictor as well. In a case report, both regional and abscopal response were obtained after combining PD-1 inhibitor and SBRT treatments in three advanced or recurrent NSCLC patients with low TMB, microsatellite stable (MSS), proficient mismatch repair (MMR), and negative PD-L1 expression (131).

The following problems could restrict the TMB evaluation in patient selection. Firstly, TMB varies in NSCLCs with different smoking histories, ages, and mutations in genes essential for DNA repair and replication (132). Secondly, the optimal methodology for TMB measurement has not been made a consensus. Secondly, a high TMB has been defined at various thresholds across different platforms, which may influence the accuracy and predictive power of TMB (132, 133). Therefore, the good cut point of TMB and the optimal gene panel size in clinical use are urgent to be defined in further studies.

### 4.5 Liquid Biopsies for Circulating Biomarkers

It is difficult to obtain enough lung biopsy in some cases, therefore, invasive biomarkers derived from peripheral blood are attractive options compared with tissue derived biomarkers. In addition, the circulating predictors could make it convenient for dynamic evaluation of patient response during treatment.

#### 4.5.1 ct-DNA

Circulating tumor DNA (ctDNA) is derived from tumor cells and includes the genomic information of tumors. It could be detected through different methods, such as NGS, qPCR and droplet digital PCR (ddPCR) (134).

Apart from monitoring tumor progression and recurrence, qualification of ctDNA level is also an effective way to monitor tumor cell death in real time. The drop in ctDNA after administration of ICI has been validated to predict responders and the prolonged survival in NSCLC, and vice versa. Notably, this biomarker changed more rapidly and profoundly than tumor size measured by imaging ways, such as CT (97–99). Another advantage of ctDNA reported by Jenny H et al. is that it could accurately discriminate tumor pseudo-progression from true progression in melanoma cohort within 12 weeks of PD-1 antibodies treatment. More surprisingly, outcomes of a prospective study in advanced conducted by Laura et al. showed that augmentation of ctDNA predicted progression after immunotherapy with 100% specificity (135).

The predictive value of ctDNA in radiotherapy is still unclear yet. It has been confirmed to increase shortly after radiation and then decrease at one week in advanced NSCLC (136, 137). They hypothesize that the ctDNA may originate from apoptotic tumor cells since higher ctDNA level was detected at 24h post radiation, which is similar to the kinetics of tumor cell apoptosis (136). However, data from relative studies is too limited to reach a consensus.

#### 4.5.2 Peripheral Blood Cells and Lymphocyte Ratios

The circulating lymphocytes are critical to the activation of systemic anti-tumor immune response and thus the efficacy of various treatment modalities. Although there are differences between the composition of lymphocytes in peripheral blood and TME, scientists hypothesize that circulating lymphocytes can reflect the immune responses in TME indirectly. In this context, multiple inflammation-related blood parameters such as white blood cells, neutrophil cells, NK cells, monocytes, platelet, MDSC and lactate dehydrogenase (LDH) have been explored for their predictive or prognostic power of ICI treatment across different cancer types, including NSCLC (88). Higher baseline median absolute lymphocyte count (ALC) significantly associates with appearance of abscopal effects post radiotherapy and improved survival benefits in patients with ICI treatment (87, 88). A larger number of proliferative PD-1^+^ CD8 T^+^ cells and active NK cells have been detected in the peripheral blood of responders that obtained better survival benefits after anti-PD-1/PD-L1 treatments (89, 90).

Inflammatory indexes such as high level of baseline neutrophil to lymphocyte ratio (NLR), platelet-to-lymphocyte ratio (PLR), and low level of lymphocyte to monocyte ratio (LMR) are reliable indicators of poor survival in advanced NSCLC in multiple retrospective pool analysis (138–140). A similar association of NLR with patient survival was also observed in radiotherapy. Both high baseline NLR and the significantly increased NLR after treatment were associated with higher mortality in SBRT treated early-stage NSCLC patients (91). Post-Stereotactic radiosurgery (SRS) NLR> 6 was a significant marker for worse OS of NSCLC with brain metastasis (92). Integrating NLR and other known biomarkers has demonstrated better predictive value for immunotherapy. Laura Mezquita et al. generated a Lung Immune Prognostic Index (LIPI) based on the derived NLR and lactate dehydrogenase (LDH) as a predictor for anti-PD-1/PD-L1 treatment through stratifying patients into three risk groups (good, 0 factors; intermediate, 1 factor; poor, 2 factors) with the poor LIPI patients having the worst survival outcome and highest risk of progressive disease (93). Shortly afterward, several studies confirmed the prognostic value of LIPI in terms of specific PD-L1 blockades, including Atezolizumab and nivolumab (94, 95). Apart from LIPI, NLR level added independent prognostic value to TMB in the context of 1714 patients across a wide range of tumor types. The chance of benefiting from ICI treatment was three times higher in NLR-low/TMB-high group than in the NLR-high/TMB-low group, which showed that this combination had a promise to improve patient selection in clinical practice (96).

#### 4.5.3 MicroRNAs

miRNAs represent single-stranded, ~22 nucleotide long non-coding RNAs. They bind to miRNA response elements (MREs), the short core sequence mainly in the 3′untranslated region (UTR) of their target messenger RNAs (mRNAs), and lead to inhibition of translation or mRNA degradation. Many miRNAs have been associated with radiosensitivity and radiotherapy efficacy by targeting different molecules and influencing various mechanisms, such as DNA damage repair, cell cycle regulation, apoptosis, glycolysis, autophagy (141–143). Great efforts have been focused on characterizing miRNA expression signatures for radiotherapy selection and patient stratification (144, 145).

Recent studies showed that multiple miRNAs could regulate PD-L1 expression. These miRNAs can affect tumor immune microenvironment and T cell activities which are pivotal to PD-1/PD-L1 blockades responsiveness. Researchers have identified several miRNA signatures that demonstrate the ability to predict clinical outcomes of NSCLC patients treated with nivolumab (146, 147). Of note, the combination of a plasma microRNA-signature classifier (MSC) and PD-L1 showed the more potent predictive value and could identify NSCLC patients with the worst clinical outcome (0% one-year PFS and OS subgroup with no favorable markers) in immunotherapy (100).

Overall, reports from accumulative studies have recognized the role of miRNA signatures as potential biomarkers to radiotherapy and immunotherapy. Compared with tumor tissue-based markers, detection of circulating miRNAs is a less invasive and more cost-effective way to monitor diseases. Circulating miRNA has advantages of stability and real-time supervision because they can be packaged in exosomes or other micro-vesicles and bind to protein complexes such as Argonaute2, which help them avoid degradation in plasma (148, 149). However, the predictive value of these miRNA signatures needs to be validated in larger NSCLC cohorts before they could be applied to clinical use.

### 4.6 Imaging Biomarkers

Tumor tissue is characterized by the high metabolism status and glycolysis rate because of the rapid proliferation of both tumor cells and lymphocytes and activation of immune responses. Chang. et al. suggested that PD-L1 enhanced glucose metabolism in tumor cells, leaving immune cells with insufficient glycolysis to maintain anti-tumor activity and proliferation, thus promoting tumor progression. While Immune checkpoint blockade reversed the tumor-induced glucose depletion in TILs and hence improving their effector function (150). The increase of metabolic parameters, such as standardized uptake value (SUV), metabolic tumor volume (MTV), and tumor lesion glycolysis (TLG) based on [18F]-fluoro-2-deoxy-d-glucose (^18^F-FDG) positron emission tomography (PET), was found to associate with a high PD-L1 level and TILs that express PD-1, CD8, CD163 (tumor-associated macrophages) and Foxp3 (Tregs) by IHC (101, 151, 152). Therefore, increasing studies have tried to validate the predictive role of tumor metabolic variables in immunotherapy (101, 102).

18F-FDG PET/CT is recognized as an essential tool in radiation treatment planning, such as the tumoral target delineation. It is valuable in predicting the outcome in less malignant lung cancer patients treated with SABR or SBRT. There seems to be a consensus that patients with high baseline SUV, MTV, and TLG are correlated with poorer clinical outcomes after radiotherapy (103–105). In addition to metabolic predictors, radiomics— a more advanced high-throughput image analysis method has been recently under investigation. It can reflect intra-tumoral histopathologic properties quantitatively and showed great power in predicting patients’ survival benefits, local recurrence, and distant metastasis after radiotherapy (106).

Generally, evaluation based on imaging tools, including PET and CT, is a good way to predict responsiveness to radiotherapy and immunotherapy, especially for patients who cannot get biopsy samples because of infection or multiple tumor lesions. Signatures integrating imaging predictors and other biomarkers, such as miRNAs signatures and dNLR, have demonstrated better choices (107, 145). However, the highly variable repeatability among those radiomic features could restrict their wide application in the future clinic (106). Many details of these imaging biomarkers, such as the optimal time for evaluation and cutoff values of these parameters, should be further defined in more prospective multicenter studies.

## 5 Discussion

Radiotherapy is one of the traditional anti-tumor treatment modalities for NSCLC. Its technological advances have allowed precise delivery of radiation to the regional tumor with an improved toxicity profile. Radiation on tumor cells can induce double strand DNA break and secretion of type I IFN through cytosolic DNA sensing pathway activation. In addition, radiotherapy stimulates anti-tumor immune responses in multiple ways, including antigen presentation enhancement, T cell priming, and T cell repertoire expansion. However, the efficacy of radiotherapy is not satisfying. Abscopal effects induced by irradiation are rare due to immunosuppressive effects caused by upregulated immune checkpoints and recruitment of immune inhibitory cells. Therefore, the combination of radiotherapy with ICIs is promising to generate synergistic immune effects. Multiple preclinical and clinical trials have evaluated patients’ responses to and clinical outcomes for immuno-radiotherapy. Generally, this combination strategy demonstrated improved response rate and survival benefits in NSCLC of different stages. However, details for this combination strategy need to be further investigated regarding the optimal dose and fraction of radiation, the sequence for radiotherapy and ICIs treatment.

Furthermore, biomarkers for predicting immune response to anti-PD-1/PD-L1 blockades and tumor radiosensitivity are essential in the era of precision medicine. It will allow clinicians to choose the most effective treatment modality for every individual and discontinue the inefficient regimens as early as possible. PD-L1 is the only biomarker widely used for patient stratification for ICI treatment in clinical practice, but its predictive role is debatable. Many other markers based on tumor tissue, peripheral blood, and radiological images have been investigated, including TMB, infiltrated lymphocytes, tumor gene signatures, ctDNA, peripheral lymphocytes, microRNAs, and imaging biomarkers. Some of these predictors can help personalize therapeutic management, such as adjusting the radiation dose prescription according to risk assessment for better efficacy and fewer injuries. In addition to biomarkers mentioned in this review, other molecules such as T cell clonality and gut microbiome have also been reported to correlate with response to immunotherapy or radiotherapy. However, all of them showed the limited capability of discriminating non-responders from responders regarding radiotherapy and immunotherapy. Moreover, the standardized cutoff points, methodologies, optimal timepoint for detection in these biomarkers have not been determined yet. In this context, the combination of different biomarkers has been proved to add accuracy to a single marker. Real-time supervision of these markers may improve their predictive values as well. Therefore, more investigations are needed to develop novel cycling markers and explore the predictive power of previously tumor tissue-based markers from periphery blood.

## Author Contributions

LM, JFX and YY have contributed to the writing of this manuscript. All authors contributed to the article and approved the submitted version.

## Funding

This research was supported by Scientific and Innovative Action Plan of Shanghai (20Y11913600), Natural Science Foundation of Shanghai (21ZR1453300), Shanghai Pulmonary Hospital (fkgg1808) and National Natural Science Foundation of China (81602657).

## Conflict of Interest

The authors declare that the research was conducted in the absence of any commercial or financial relationships that could be construed as a potential conflict of interest.

## Publisher’s Note

All claims expressed in this article are solely those of the authors and do not necessarily represent those of their affiliated organizations, or those of the publisher, the editors and the reviewers. Any product that may be evaluated in this article, or claim that may be made by its manufacturer, is not guaranteed or endorsed by the publisher.
